# Left atrial appendage morphology with the progression of atrial fibrillation

**DOI:** 10.1371/journal.pone.0278172

**Published:** 2022-11-30

**Authors:** Yoichi Takaya, Rie Nakayama, Fumi Yokohama, Norihisa Toh, Koji Nakagawa, Masakazu Miyamoto, Hiroshi Ito

**Affiliations:** Department of Cardiovascular Medicine, Okayama University Graduate School of Medicine, Dentistry and Pharmaceutical Sciences, Okayama, Japan; Ohio State University, UNITED STATES

## Abstract

Left atrial appendage (LAA) size is crucial for determining the indication of transcatheter LAA closure. The aim of this study was to evaluate the differences in LAA morphology according to the types of atrial fibrillation (AF). A total of 299 patients (mean age: 67 ± 13 years) who underwent transesophageal echocardiography (TEE) were included. Patients were classified into non-AF (n = 64), paroxysmal AF (n = 86), persistent AF (n = 87), or long-standing persistent AF (n = 62). LAA morphology, including LAA ostial diameter and depth, was assessed using TEE. Patients with long-standing persistent AF had larger LAA ostial diameter and depth and lower LAA flow velocity. The maximum LAA ostial diameter was 19 ± 4 mm in patients with non-AF, 21 ± 4 mm in patients with paroxysmal AF, 23 ± 5 mm in patients with persistent AF, and 26 ± 5 mm in patients with long-standing persistent AF. LAA ostial diameter was increased by 2 or 3 mm with the progression of AF. LAA ostial diameter was correlated with LA volume index (R = 0.37, P < 0.01) and the duration of continuous AF (R = 0.30, P < 0.01), but not with age or the period from the onset of AF. In conclusion, LAA size was increased with the progression of AF.

## Introduction

Atrial fibrillation (AF) is caused by left atrial (LA) dilatation. The presence of AF further promotes LA remodeling, leading to LA enlargement. LA dilatation is enhanced by the progression of AF [1]. Patients with AF are at increased risk of cardioembolic stroke [2]. LA appendage (LAA), which is a finger-like extension of the left atrium with various morphologies, is the origin of embolism in more than 90% of patients with AF [3]. Recently, transcatheter closure of LAA with the Watchman device (Boston Scientific, Marlborough, Massachusetts, USA) has been proposed as an effective therapy for prevention of embolization [4]. The assessments of LAA morphology using transesophageal echocardiography (TEE) are crucial for determining the indication of transcatheter LAA closure. LAA size has been reported to be increased in patients with AF [5–7]. However, the relationships of LAA size with the types of AF have not been well investigated. The aim of this study was to evaluate the differences in LAA morphology according to the types of AF.

## Methods

### Study population

We enrolled a total of 299 patients who underwent TEE in our institution from October 2019 to October 2020. Patients with atrial septal defect and those with degenerative mitral regurgitation (MR) were excluded. In this study, patients were classified into non-AF, paroxysmal AF defined as continuous AF for <7 days in duration, persistent AF defined as continuous AF for ≥7 days and <1 year in duration, or long-standing persistent AF defined as continuous AF for ≥1 year in duration. All patients provided written informed consent for examination. This study was in accordance with the Declaration of Helsinki and approved by the ethics committee of Okayama University Hospital.

### Transesophageal echocardiography

**Fig 1:**
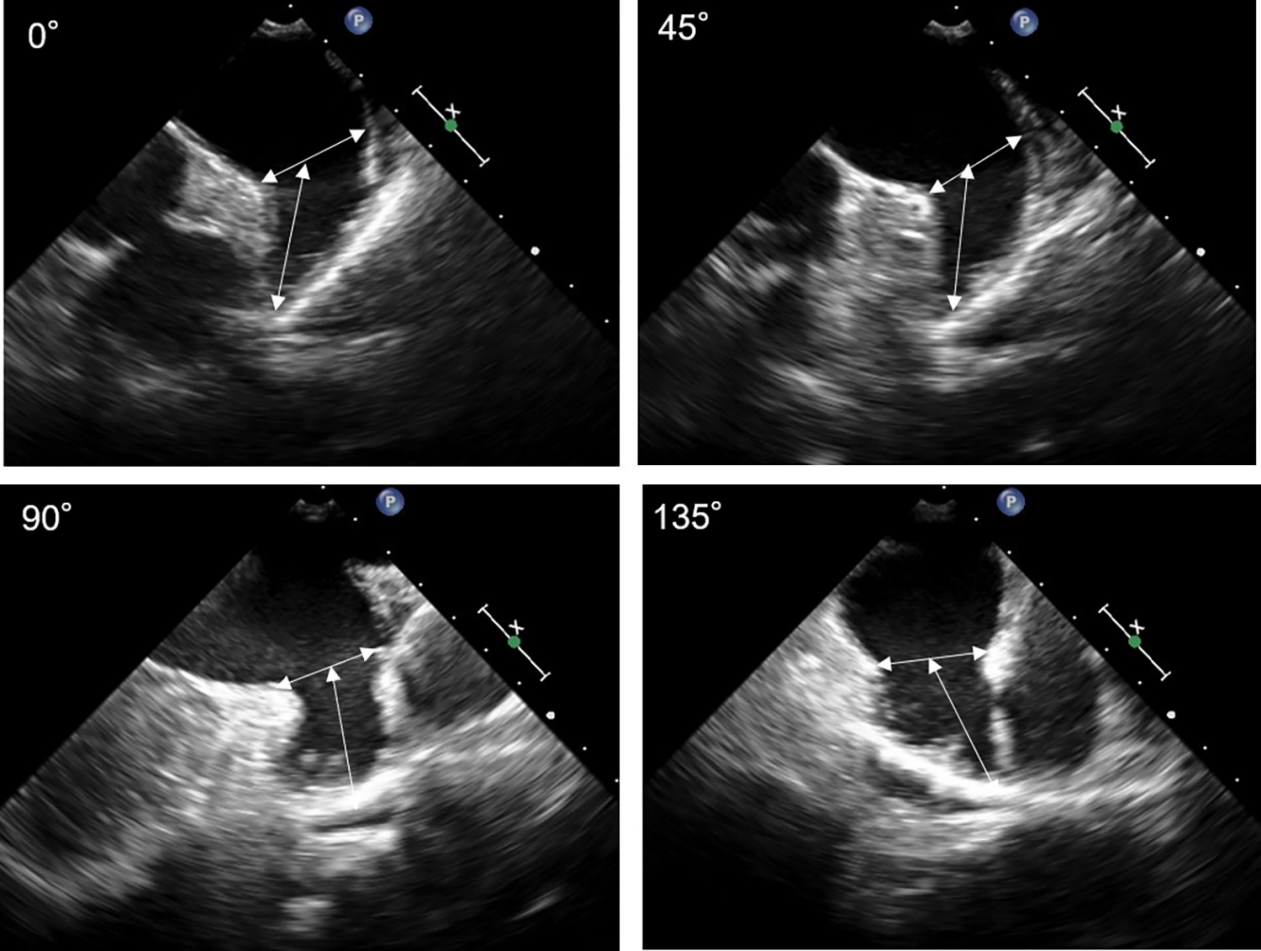
Transesophageal echocardiography measurement of left atrial appendage size. Left atrial appendage ostial diameter and depth were measured at 0°, 45°, 90°, and 135°.

TEE (iE33 with an X7-2t probe; Philips Medical Systems, Best, The Netherlands) was performed by experienced cardiologists. All images were digitally stored. TEE images of LAA were obtained at an angle of 0°, 45°, 90°, and 135° views, based on the strategy of transcatheter LAA closure with the Watchman device. LAA ostial diameter was measured in each view from the level of the circumflex artery to the appropriate location of the device implantation along the coumadin ridge. LAA depth was measured in each view from the ostium to the tip of LAA [8] (Fig 1). When the circumflex artery was not visible, especially at TEE view of 135°, LAA ostial position was identified by referring to the distance from the probe to the ostium in TEE views of other angles. The measurements of LAA ostial diameter and depth were performed at the end-ventricular systole. In patients with AF, we measured three times and selected the maximum value. Then, the maximum LAA ostial diameter and depth among TEE views of 0°, 45°, 90°, and 135° were determined. The shape of LAA was classified into chicken wing or non-chicken wing, including windsock, cauliflower, and cactus [9]. LAA flow velocity was measured with pulsed wave Doppler imaging in the proximal third of the appendage.

### Clinical assessments

CHA_2_DS_2_-VASc (congestive heart failure, hypertension, age ≥75 years, diabetes mellitus, prior stroke or transient ischemic attack or thromboembolism, vascular disease, age 65–74 years, sex category) and HAS-BLED (hypertension, abnormal renal or liver function, stroke, bleeding, labile international normalized ratio, elderly, drugs or alcohol) scores were calculated. The duration of continuous AF and the period from the onset of AF were estimated by symptoms and electrocardiographic findings. For example, in a case of paroxysmal AF which continued for several days from one year ago, we determined that the duration of continuous AF was several days and the period from the onset of AF was one year. Transthoracic echocardiography was performed before TEE examination. Left ventricular (LV) end-diastolic and end-systolic diameters, LV ejection fraction, LA diameter, LA volume index, early diastolic mitral valve flow velocity (E), early diastolic septal mitral annular velocity (e’), the degrees of MR and tricuspid regurgitation (TR), and TR pressure gradient were obtained. LA diameter was measured at the end-ventricular systole on the parasternal long-axis view. LA volume was derived using the biplane Simpson’s technique on the apical four-chamber and two-chamber views, and was normalized for body surface area.

### Statistical analysis

Continuous variables are presented as mean ± standard deviation. Categorical variables are presented as number and percentage. Differences between groups were analyzed using the 1-way ANOVA for continuous variables and using the χ^*2*^ test or the Fisher exact test for categorical variables. Pearson’s correlation coefficient was used to assess correlations between variables. Statistical analysis was performed with JMP version 14.0 (SAS Institute Inc., Cary, NC, USA), and significance was defined as a P value of < 0.05.

Interobserver and intraobserver differences were analyzed among 50 randomly selected cases. LAA ostial diameter was measured by two blinded observers and by a single observer at two different times. Reliability was calculated using Pearson’s correlation coefficient. Variability was calculated as the percentage error of each measurement and derived as the difference between the measurements divided by the mean value.

## Results

### Patient characteristics

The mean age of all patients was 67 ± 13 years, and 192 patients were male. Patient characteristics and echocardiographic parameters are shown in Table 1. Sixty-four patients had non-AF, 86 had paroxysmal AF, 87 had persistent AF, and 62 had long-standing persistent AF. Of the 64 patients with non-AF, 11 underwent TEE for assessing aortic stenosis or aortic regurgitation, 12 for assessing MR, 1 for assessing TR, and 40 for investigating the cause of cerebral embolism such as patent foramen ovale. During echocardiographic examinations, of the 86 patients with paroxysmal AF, 80 had sinus rhythm and 6 had AF. Of the 87 patients with persistent AF, 26 had sinus rhythm and 61 had AF.

**Table 1:** Patient characteristics and echocardiographic parameters.

	Non-AF (n = 64)	Paroxysmal AF (n = 86)	Persistent AF (n = 87)	Long-standing persistent AF (n = 62)	P
Age, years	59 ± 16	69 ± 11	68 ± 12	70 ± 11	< 0.01
Male	42 (66%)	50 (58%)	56 (64%)	44 (71%)	0.44
Body surface area, m^2^	1.6 ± 0.2	1.6 ± 0.2	1.7 ± 0.2	1.6 ± 0.2	0.21
CHA_2_D_2_-VASc score	1.7 ± 1.8	2.8 ± 1.5	3.3 ± 1.8	3.8 ± 2.0	< 0.01
HAS-BLED score	0.6 ± 0.8	1.4 ± 1.1	1.2 ± 0.9	1.7 ± 1.3	< 0.01
LV end-diastolic diameter, mm	47 ± 7	47 ± 8	47 ± 7	47 ± 6	0.99
LV end-systolic diameter, mm	31 ± 7	32 ± 8	33 ± 8	33 ± 7	0.41
LV ejection fraction, %	62 ± 8	60 ± 12	56 ± 13	56 ± 12	0.01
LA diameter, mm	37 ± 7	40 ± 6	45 ± 5	46 ± 7	< 0.01
LA volume index, ml/m^2^	39 ± 15	41 ± 10	52 ± 16	60 ± 18	< 0.01
E, cm/s	63 ± 23	68 ± 20	83 ± 25	92 ± 35	< 0.01
e’, cm/s	6 ± 2	5 ± 2	7 ± 2	6 ± 2	< 0.01
E/e’ ratio	12 ± 9	14 ± 6	14 ± 7	17 ± 9	< 0.01
Moderate-to-severe MR	8 (13%)	7 (8%)	23 (26%)	19 (31%)	< 0.01
Moderate-to-severe TR	4 (6%)	10 (12%)	20 (23%)	17 (27%)	< 0.01
TR pressure gradient, mm Hg	26 ± 19	25 ± 7	26 ± 9	29 ± 10	0.24

Data are presented as mean ± standard deviation or number (%) of patients.

Abbreviations: AF, atrial fibrillation; CHA_2_D_2_-VASc, congestive heart failure, hypertension, age ≥75 years, diabetes mellitus, prior stroke or transient ischemic attack or thromboembolism, vascular disease, age 65–74 years, sex category; E, early diastolic mitral valve flow velocity; e’, early diastolic septal mitral annular velocity; HAS-BLED, hypertension, abnormal renal or liver function, stroke, bleeding, labile international normalized ratio, elderly, drugs or alcohol; LA, left atrial; LV, left ventricular; MR, mitral regurgitation; TR, tricuspid regurgitation.

Patients with long-standing persistent AF had larger LA dilatation and LA volume index, higher E/e’ ratio, and moderate-to-severe MR and TR more frequently. CHA_2_DS_2_-VASc and HAS-BLED scores were higher in patients with long-standing persistent AF.

### Left atrial appendage morphology

LAA ostial diameter and depth according to the types of AF are shown in Table 2. In TEE views of an angle of 0°, 45°, 90°, and 135°, LAA ostial diameter and depth were larger in patients with long-standing persistent AF. The maximum LAA ostial diameter measured in any TEE views of 0°, 45°, 90°, or 135° was 19 ± 4 mm in patients with non-AF, 21 ± 4 mm in patients with paroxysmal AF, 23 ± 5 mm in patients with persistent AF, and 26 ± 5 mm in patients with long-standing persistent AF. LAA ostial diameter was increased by 2 or 3 mm with the progression of AF. Patients with long-standing persistent AF had lower LAA flow velocity. LAA shape was not different in the types of AF.

**Table 2:** Left atrial appendage morphology according to the types of atrial fibrillation.

	Non-AF (n = 64)	Paroxysmal AF (n = 86)	Persistent AF (n = 87)	Long-standing persistent AF (n = 62)	P
LAA ostial diameter, mm					
0°	15 ± 5	18 ± 4	20 ± 5	22 ± 5	< 0.01
45°	15 ± 4	17 ± 4	19 ± 4	21 ± 4	< 0.01
90°	15 ± 4	17 ± 4	19 ± 4	22 ± 4	< 0.01
135°	18 ± 4	20 ± 5	22 ± 5	25 ± 5	< 0.01
Maximum	19 ± 4	21 ± 4	23 ± 5	26 ± 5	< 0.01
LAA depth, mm					
0°	25 ± 6	28 ± 6	30 ± 7	30 ± 7	< 0.01
45°	25 ± 6	28 ± 6	29 ± 6	31 ± 6	< 0.01
90°	24 ± 6	27 ± 6	29 ± 6	31 ± 7	< 0.01
135°	23 ± 6	27± 6	29 ± 7	31 ± 7	< 0.01
Maximum	28 ± 9	30 ± 6	33 ± 6	34 ± 7	< 0.01
LAA shape					
Chicken wing	17 (27%)	21 (24%)	15 (17%)	10 (16%)	0.33
Non-chicken wing	47 (73%)	65 (76%)	72 (83%)	52 (84%)	
LAA flow velocity, m/s	55 ± 19	50 ± 19	32 ± 19	26 ± 15	< 0.01

Data are presented as mean ± standard deviation or number (%) of patients.

Abbreviations: AF, atrial fibrillation; LAA, left atrial appendage.

### Correlation with left atrial appendage size

**Fig 2:**
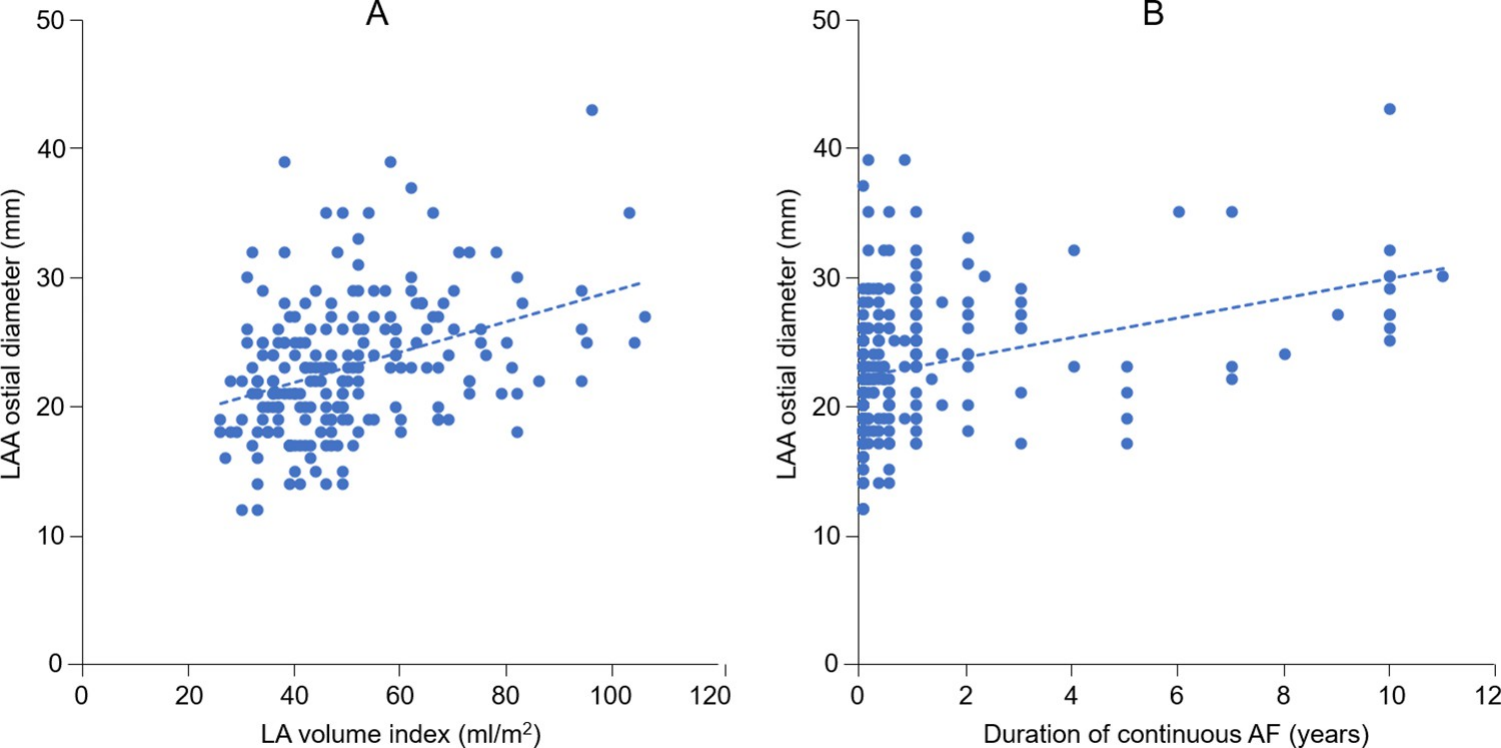
Relationships of left atrial appendage ostial diameter with left atrial volume index (A) and the duration of continuous atrial fibrillation (B). Abbreviations: AF, atrial fibrillation; LA, left atrial; LAA, left atrial appendage.

Among patients with paroxysmal AF, persistent AF, and long-standing persistent AF, LAA ostial diameter (R = 0.37, P < 0.01) and LAA depth (R = 0.44, P < 0.01) were correlated with LA volume index. LAA ostial diameter was correlated with the duration of continuous AF (R = 0.30, P < 0.01) (Fig 2), but not with age or the period from the onset of AF. The correlations of LAA ostial diameter with CHA_2_D_2_-VASc or HAS-BLED scores were not observed.

### Variability

There was good agreement in the measurements of LAA ostial diameter in the interobserver assessment (R = 0.92, P < 0.01) and intraobserver assessment (R = 0.93, P < 0.01). The interobserver and intraobserver variabilities in LAA ostial diameter were 4.9% and 4.3%, respectively.

## Discussion

AF is the most common cardiac arrhythmia, which increases in prevalence by aging. LA dilatation is well known to be related to an increase in burden of AF [1]. Regarding LAA, some studies reported that LAA size was larger in patients with persistent AF [5–7]. One study reported a progressive increase in LAA orifice area with increasing frequency of AF [10]. However, whether LAA morphology, including LAA ostial diameter and depth, is affected by the progression of AF (non-AF, paroxysmal AF, persistent AF, long-standing persistent AF) remains unclear.

The present study evaluated the differences in LAA morphology according to the types of AF and showed that patients with long-standing persistent AF had larger LAA ostial diameter and depth. LAA ostial diameter was increased with the progression of AF (from non-AF to long-standing persistent AF), and was correlated with the duration of continuous AF. LAA has higher compliance compared with LA wall. LAA assists with the adjustability of LA pressure [11]. Therefore, elevated LA pressure due to the persistence of AF may cause the enlargement of LAA ostial diameter [12,13]. Additionally, LAA ostial diameter was correlated with LA volume, indicating that LA dilatation may also stretch LAA ostial diameter.

LAA is a site of thrombus formation. LAA removal or occlusion is performed to reduce the risk of stroke in patients with AF undergoing open heart surgery [14]. Recently, the effectiveness of transcatheter closure of LAA with the occlusion device has been demonstrated in patients with AF ineligible to anticoagulation therapy. With the advent of transcatheter LAA closure, LAA morphology has become the focus of increasing interest. To implant the occlusion device, such as the Watchman device, LAA ostial diameter and depth are important for selecting the appropriate device size. The present study showed that the maximum LAA ostial diameter was increased by 2 or 3 mm as AF progressed. This result indicates that larger sized devices are required in patients with persistent and long-standing persistent AF.

Several studies have reported that large LAA size is at risk of stroke [15,16]. The increased number of LAA lobes has been reported to be associated with thrombus formation [17]. The reduced LAA flow velocity is established to be related to an increased risk of thrombus formation [18,19]. In this study, patients with persistent and long-standing persistent AF had larger LAA size and lower LAA flow velocity. Therefore, transcatheter LAA closure may have greater benefits for patients with persistent and long-standing persistent AF.

The present study has several limitations. First, the number of patients was relatively small. Larger study population is needed to confirm our findings. Second, this study included patients who underwent TEE. Patients with non-AF were not healthy subjects. Third, the duration of continuous AF might be underestimated because AF is a disease of partially silent nature. Fourth, echocardiographic data might not be accurate because patient’s rhythm was not uniform during the examinations. Finally, LAA morphology was not assessed by 3-dimensional TEE. LAA ostial diameter and depth might not be strictly accurate.

In conclusion, LAA size was increased with the progression of AF. LAA ostial diameter was correlated with the duration of continuous AF.

## Supporting information

S1 Data (XLSX)
